# Impact of the FLS model on patients with major fracture in Gran Canaria: 2018–2022 experience

**DOI:** 10.1007/s11657-025-01514-7

**Published:** 2025-03-07

**Authors:** Antonio Naranjo, Cristian Sarmiento, Amparo Molina, Sonia Fuentes, Laura Cáceres, Soledad Ojeda

**Affiliations:** 1 Rheumatology Department, Hospital Universitario de Gran Canaria Dr. Negrín, Barranco de La Ballena, 35011 Las Palmas de Gran Canaria, Spain; 2 University of Las Palmas de Gran Canaria, Las Palmas de Gran Canaria, Spain

**Keywords:** Fracture Liaison Service, Osteoporosis, Fracture

## Abstract

***Summary*:**

We analyzed 5396 patients with fragility fracture, their inclusion by the FLS, and prescription of treatment. Thirty-four percent of potential cases were attended by the FLS, and at the healthcare level, the impact of FLS model resulted in an increase of treated patients from 20% in standard care to 41%.

**Introduction:**

Patients with fragility fractures are at high risk of new fractures, with a negative impact on their quality of life, as well as higher mortality and costs for the health system, especially for hip fractures. Less than 20% of patients receive treatment (lifestyle advice, calcium, vitamin D, and bisphosphonate) after a fracture. The fracture liaison service (FLS) is the most effective model for secondary prevention.

**Objectives:**

To analyze the incidence of fragility fractures in the area of Gran Canaria North and the impact of the FLS unit on the prevention of new fractures.

**Methods:**

Patients > 50 years were attended at the emergency department for fractures of the proximal femur, proximal humerus, distal forearm, pelvis, or vertebra during the period 2018–2022 were included. A file was constructed containing demographic data, type of fracture, inclusion in the FLS, and the initiation of treatment to prevent new fractures. A sample of patients not treated at the FLS was selected for estimating the prophylaxis of fractures under standard care management.

**Results:**

A total of 5396 patients were included, 74.2% women, with a mean age of 74 years. After excluding 558 traumatic fractures (10.3%), 318 (5.9%) deaths, and 167 (3.1%) cases due to a lack of criteria, the sample of potential patients who were candidates for FLS was 4353. This represented 80.6% of the initial sample, of which 1497 patients (34.4%) were attended at the unit. Factors independently associated with referral to the FLS were younger age (OR 0.97; 95% CI 0.97–0.98), female sex (OR 2.24; 95% CI 1.91–2.61), and humerus fracture (OR 1.34; 95% CI 1.16–1.55). Treatment to prevent fractures was verified in 1189 patients (79.4%) in the FLS group and in 585 (20.4%) of those with fragility fractures who were not included. At the healthcare level, the services provided by the FLS resulted in an increase in treated patients from 20% in standard care to 41% with the FLS model.

**Conclusions:**

In terms of treatment initiation to new fracture prevention at the healthcare level, the FLS achieved a twofold increase. The high incidence of fractures and the progressive aging of the population underline the effectiveness of the FLS secondary prevention model.

## Introduction

Osteoporosis is a systemic skeletal disease characterized by low bone mass and deterioration in the microarchitecture of bone tissue, with a consequent increase in bone fragility and susceptibility to fracture [1]. Age and female gender, as well as other clinical risk factors, in a summative form, were predictive of patients who were at high risk of osteoporosis and fracture. The fracture risk assessment tool, FRAX®, provides specific algorithms to estimate the 10-year risk of hip fracture and major osteoporotic fractures based on a series of clinical risk factors [2].

Fragility fractures are those produced by an impact that would be insufficient to fracture a normal bone, for example, fractures that occur due to a patient’s height. This often represents a major clinical manifestation of osteoporosis. Fragility fractures are more common in patients over 50 years of age and in those who have suffered previous fractures. These fractures have relevant morbidity and mortality consequences for patients and the health system, especially in the case of a hip fracture.

Osteoporosis is the most common metabolic bone disease in western countries, affecting 25–32% of women over 50 years of age and almost 50% over 75 years of age [1]. In Spain, a total of 330,000 fractures occur every year, an incidence that is increasing due to the progressive aging of the population. An increase of around 30% in the number of fragility fractures is estimated by 2034, reaching 370,000 cases annually [3]. The total healthcare cost associated with fragility fractures in Spain was, in 2019, 4.3 billion euros, 3.8% of healthcare spending, above the European average. It is estimated that by 2030, health expenditures associated with fractures will reach 5.5 billion euros [3].

The most common fractures are vertebral fractures, with an estimated prevalence between 25 and 50% in people over 50 years of age [4–7]. Usually located in the dorsal and lumbar region, vertebral fractures can cause acute pain or be asymptomatic, which explains why they are frequently underdiagnosed. The most serious osteoporotic fracture is hip fracture, with a 1-year mortality rate of approximately 20% [8].

Bisphosphonates are the standard treatment for osteoporosis, as they are safe, effective, and convenient [9]. Although their use has achieved a 22% reduction in the incidence of hip fractures [10], less than 20% of patients receive antiresorptive treatments such as bisphosphonates after sustaining a fragility fracture [11, 12]. Current guidelines not only include bisphosphonates and denosumab for patients with high and very high risk of fracture, but also anabolic drugs and sequential therapies.

A “Fracture Liaison Service,” or FLS, is a secondary fracture prevention unit that cares for patients who have suffered a fragility fracture, providing multidisciplinary care, assessments, and treatments according to established guidelines [13, 14]. The implementation of FLS in the northern area of Gran Canaria has led to an increase in treatments to prevent new fractures [15, 16], similar to the results achieved in other countries [17, 18]. An additional problem in the field of osteoporosis is poor adherence to treatment [19]. The FLS model has been shown to improve treatment adherence, with persistence rates of around 46.5% at 5 years [20].

The objective of the present study was to analyze the impact of the FLS model on a healthcare area, that is, the impact on treatment prescriptions to prevent fractures versus standard care management.

## Methods

### Design

To determine the incidence of fracture in an area of Gran Canaria North, a retrospective study was carried out. Patients older than 50 years who suffered a fragility fracture during the period 2018–2022 and who had been identified in the emergency registry with electronic coding ICD-10 were included. Patients identified in other hospitals or primary care were not included. Duplicate cases that involved an emergency room consultation for the same reason were considered once. In addition, of those patients who experienced a refracture during the study, only the worst type of fracture was selected as the index fracture.

The study’s principal results are presented in terms of the number of patients with a fracture and the type of fracture. Once the sample was selected, we analyzed the patients identified and evaluated by the FLS unit, which has a database dating from 2012 [15]. Assessment and follow-up by the FLS unit utilize a prospective cohort design. Patients are recruited from the emergency registry and are cared for by nurses and doctors with the first treatment order made by the FLS. After the baseline visit, most patients are followed up by telephone consultations conducted by a nurse, while others are referred to a rheumatology metabolic clinic for additional study or because of an indication of zoledronic or teriparatide. The data from the FLS database were cross-referenced with the emergency fracture records, and the following parameters were recorded: inclusion in the study, cause of non-inclusion, and treatment orders during the first 6 months after the visit to the FLS.

The prescription of treatment to prevent fractures in the FLS was collected in all patients at the baseline visit and in the first 6 months of follow-up. In addition, the electronic prescription of a random sample of 100 patients not treated at the FLS was reviewed.

### Inclusion and exclusion criteria

Patients of both sexes over 50 years who had been identified during treatment at the emergency department in the years 2018, 2019, 2020, 2021, and 2022.

Fracture of the proximal femur, proximal humerus, distal radius, pelvis, or vertebra.

We excluded patients with traumatic fractures or metastatic bone disease. For patients who suffered more than one fracture during the study period, we choose as the index fracture the most severe one following this order of descending importance: hip, vertebra, pelvis, humerus, and forearm.

### Ethical aspects

The study was approved by the local ethics and research committee.

### Statistical analysis

The results are presented using descriptive statistics: mean, median, and standard deviation. Taking into account the study objectives, the data analyses focused on the following parameters: incidence of fracture in patients over 50 years of age in the northern area of Gran Canaria, overall, by type of fracture, by sex, by age (grouped by decade), and by month of the year. The reference population was taken from the health area report [21].

To analyze the differences between qualitative variables, contingency tables and Fisher’s exact test were used, while for quantitative variables, the Student *t* test or the ANOVA analysis of variance test was used, as appropriate. A binary logistic regression model was applied if at least two variables were found to be significant in the bivariate analysis using IBM® SPSS version 27.

## Results

**Fig. 1:**
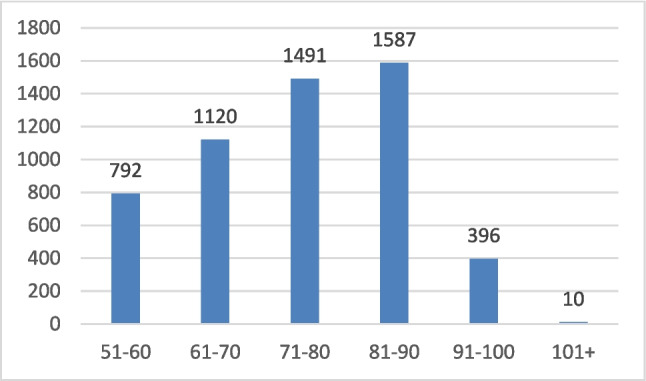
Number of patients with a fracture per decade of age

A total of 5396 patients (4005 women and 1391 men) with a fragility fracture of the hip, dorsal/lumbar vertebra, humerus, pelvis, and/or distal forearm were included. The average age of the patients identified in the study period was 74.9 years, 75.5 for women, and 73.2 for men (*p* < 0.001). Figure 1 shows the distribution of patients by age, revealing an increase for each decade up to 90 years of age.

The most common fracture in both sexes was hip fracture. For all types of fractures, the incidence was higher in women. Table 1 shows the distribution of fracture type by sex and Table 2 the average age for each type of fracture. In women, fractures of the forearm were more common than in men, while fractures of the hip and vertebra were more common in men.

**Table 1:** Type of fracture and sex of the patient

	Total *N* (%)	Women *N* (%)	Men *N* (%)	*p*
Hip	1945 (36.0)	1363 (34.0)	582 (41.8)	< 0.01
Distal forearm	1498 (27.7)	1192 (29.7)	306 (22.0)	< 0.01
Humerus	1117 (20.7)	865 (21.6)	252 (18.1)	< 0.01
Vertebra	484 (8.9)	318 (7.9)	166 (11.9)	< 0.01
Pelvis	352 (6.5)	267 (6.6)	85 (6.1)	0.46

**Table 2:** Average age for each type of fractures. SD, standard deviation

	Mean age	SD	*p*
Hip	80.2	10.2	< 0.001
Distal forearm	68.9	10.9	
Humerus	72.7	10.5	
Vertebra	74.0	10.7	
Pelvis	79.4	10.1	

**Fig. 2:**
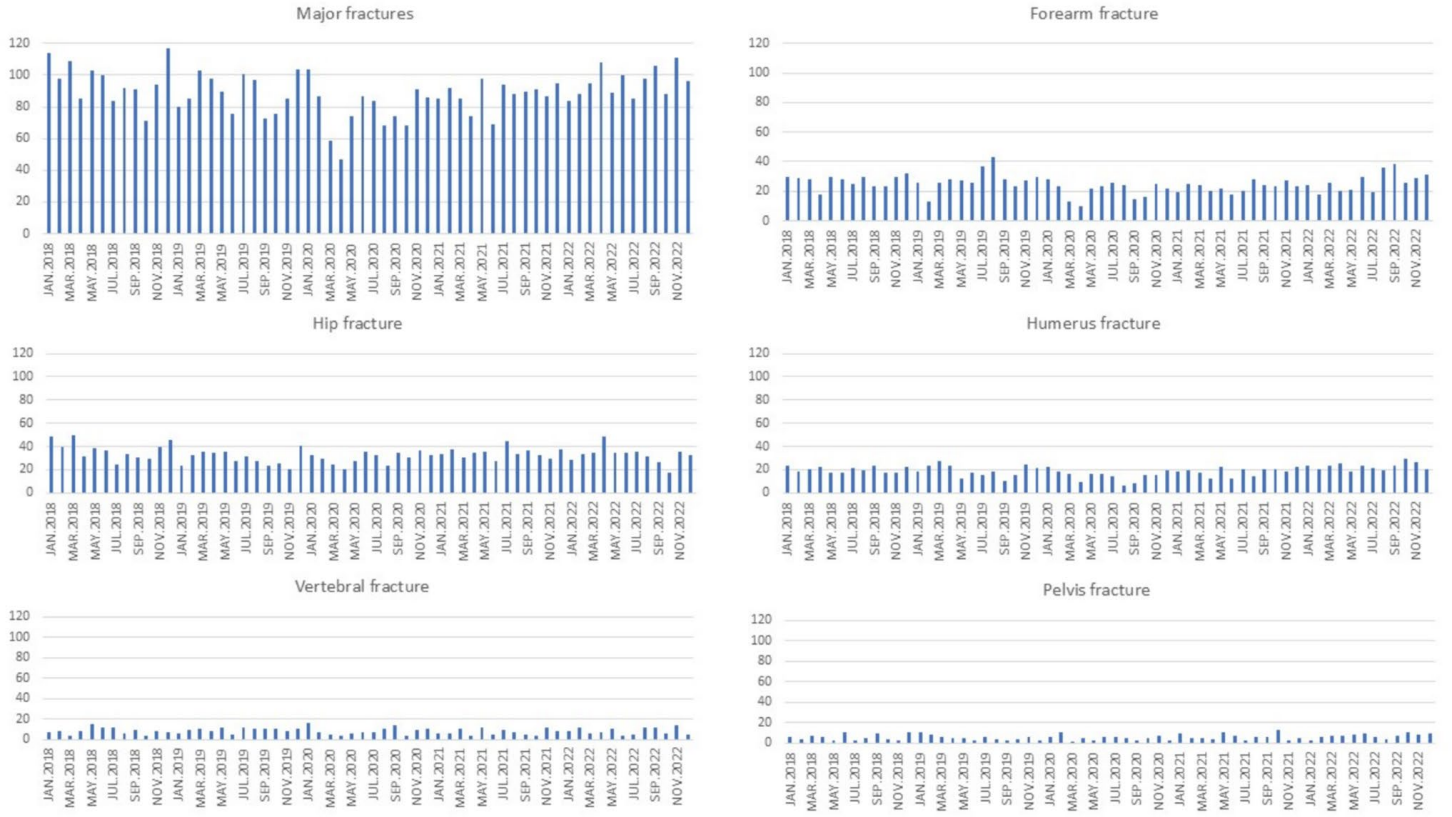
Distribution of patients with a fracture during the years 2018–2022. The bars represent the monthly number of fractures

Figure 2 shows the incidence of fracture throughout the study period. An increase in the number of fractures occurred during the winter months (*p* = 0.028 comparing the months of December and January versus the rest of the months of the year) and a decrease between March and May 2020, coinciding with the state-ordered confinement due to the Covid-19 pandemic in Spain.

### Incidence of hip fracture

The crude incidence rate of hip fracture in our geographic area in persons 50 years old or older during the study period was 207 cases per 100,000 inhabitants (275 in women and 131 in men). In people 65 years of age or older, the incidence was 455 cases per 100,000 inhabitants (587 in women and 288 in men).

### Causes of non-referral to the FLS unit

**Fig. 3:**
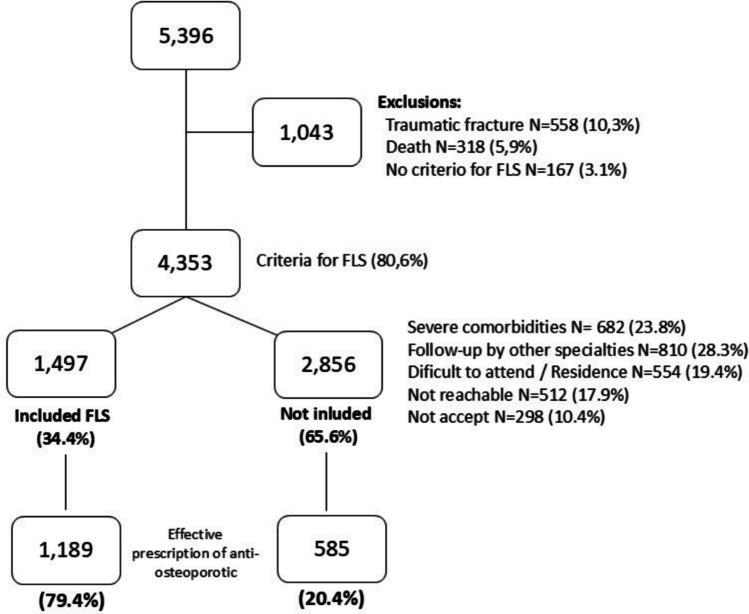
Flowchart showing patient breakdown and reasons for exclusion

The analysis of the causes of non-referral to the fracture unit was carried out with a sample of 290 patients treated in the emergency room during the last quarter of 2022. The causes for non-referral are detailed in Fig. 3, the most frequent being an untraceable patient or one who refused enrollment.

A total of 558 cases of traumatic fracture (10.3%) and 318 (5.9%) deaths were excluded; death was the most frequent cause of exclusion in hip fracture followed by pelvic fracture. In the case of exclusion due to high-intensity trauma, fractures of the forearm, humerus, and vertebra stand out in that order. Thus, the sample of potential FLS candidate patients was 4353 (Fig. 3).

### Assessment by the Fracture Liaison Service

Of the patients who were candidates for FLS, 34.4% were evaluated by the unit (38.4% of women and 21.4% of men; *p* < 0.001). More patients with fractures of the upper limb versus hip were studied in the FLS (Table 3).

**Table 3:** Patients evaluated by FLS by fracture type once traumatic fractures and deaths were excluded

		Assessed by the FLS		
	Total	No	Yes	%
Hip	1541	1068	473	30.7
Distal forearm	1220	732	488	40.0
Humerus	904	524	380	42.0
Vertebra	400	276	124	31.0
Pelvis	288	256	32	11.1

The patients who attended the FLS had a mean age of 72 years and 85% were women. The most frequent risk factors were previous fracture (18.5%), early menopause (16%), active smoking (12%), and corticosteroids (4.5%). Ten percent of patients had a treatment to prevent fractures such as bisphosphonate or similar in the FLS baseline visit. The bone densitometry results were osteoporosis in 43% of patients and osteopenia in 44%.

The mean age of those who were treated at the unit was lower than that of patients who were not included (72.8 years vs. 75.7 years; *p* < 0.001).

In a binary logistic regression analysis, in which the dependent variable was being evaluated by the fracture unit, the following factors were significantly and independently associated: younger age (OR 0.97; 95% CI 0.97–0.98), female sex (OR 2.24; 95% CI 1.91–2.61), and humerus fracture (OR 1.34; 95% CI 1.16–1.55).

### Impact of FLS in the healthcare area and refractures

Treatment to prevent fractures was initiated in 1189 patients (79.4%) who were treated at the FLS and 585 (20.4%) who were not attended there (Fig. 3). In this way, the presence of FLS in our health area adds more than 1000 patients in treatment to prevent fracture, with which the overall percentage of patients treated increases from 20.4% in standard care to 40.7% with the implementation of the FLS.

Two hundred eighty-six patients (5.3% of the initial sample) suffered a new fracture during the period analyzed, distributed as follows: 46% femur, 19% forearm, 16% humerus, 8% vertebra, and 9% other fractures.

## Discussion

In the present work, a very large cohort of patients was reviewed (involving the screening of nearly 10,000 emergency visits). In fact, it can be regarded as a closely representative snapshot of the impact of fragility fractures in the northern area of Gran Canaria. The type of fracture (the most common were of the hip and forearm), the predominance of women (2.8 for every man), and the highest frequency in the winter months (osteoporotic fractures) were among the study’s findings already published [22].

The number of vertebral fractures was low in our study, due to the fact that many such fractures are not treated by emergency departments, but rather in outpatient clinics. Vertebral fractures are frequently diagnosed as incidental radiological findings or go unrecognized, and a substantial proportion of patients consequently do not receive appropriate medical attention [23]. Our FLS does treat outpatient vertebral fractures and those identified through VFA, although these are not part of the present work.

The reduction in the number of fractures during the Covid-19 confinement (March–April) 2020 in Spain revealed by our analysis has been previously reported [24].

The Canary Islands have one of the lowest hip fracture rates in Spain [25]. The crude incidence of hip fracture per 100,000 people/year in people 65 years old or older was 455 in our study, compared to the average of 500 typically described for Spain [26]. In people aged 50 or over, the incidence in our study was 207 (275 in women and 131 in men), having published for the period 2007–2011 an average incidence in Gran Canaria of 181 cases (246 in women and 108 in men) [27].

The FLS model has been shown to not only reduce the incidence of new fractures, but also to be cost-effective [28]. The FLS model increases treatment initiation during the first 6 months after the initial visit to the unit, which could be verified via electronic prescription compared to the prescription in standard management [19, 20, 30]. In addition, adherence to osteoporosis treatment, usually low, improved substantially with the FLS model of secondary prevention [19, 29].

Despite the fact that the FLS included less than 50% of candidate patients with fragility fractures in our healthcare area, its impact on preventing new fractures was significant: double (initiation of treatment in 40.7% vs 20.4% in standard care). FLS has other added advantages, such as improving medium- and long-term adherence to osteoporosis treatment and providing patients with the appropriate diet and physical exercise regimens to prevent falls and new fractures. On the other hand, the FLS at the Dr. Negrín Hospital is the first in Spain to be proven cost-effective compared to standard management [31].

The percentage of patients finally studied and treated from those cases identified by the FLS was not very high. There are various circumstances and reasons why patients with fragility fracture are excluded or do not agree to participate in an FLS program. Thus, in several FLS-oriented studies from France, Greece, and the USA, only 29 to 31% of candidate patients were included [30–34], a rate similar to our own study. The results of our study offer insight into the real impacts an FLS can make. Certainly, the percentage of patients included can be improved. We believe that one way to increase this percentage lies in the accurate diagnosis and treatment orders for hip fractures during hospital admission, as we observed in one of our previous studies [29]. In this sense, the National Hip Database of the UK, in its 2022 report encompassing 169 hospitals, reported an average rate of treatment orders for osteoporosis of 52% [35]. For hip fractures identified during admission, it is plausible to achieve up to 70% secondary prevention. However, with an FLS that includes all major fractures, in our opinion, and taking into account the various reasons for non-inclusion, the overall number of treated fractures would likely not exceed more than 60% of those patients identified in emergency registries.

Although traumatic fractures are systematically excluded in FLS units, it has been reported that the risk of new fractures is greater than in the general population, and the risk after traumatic fracture is approximately half that of a fragility fracture [34]. In our study, around 10% of major fractures were traumatic, and studies have been published in which up to 16% of the fractures treated at an FLS were the result of non-mild trauma [36].

In a Spanish retrospective observational study (covering the years 2014 and 2015), the incidence of subsequent fractures was 6.6%, compared to 5.3% in our study (covering 2018 to 2022) [37].

Despite the good results achieved by the FLS at Dr. Negrín Hospital, a pioneering unit in Spain, we found several aspects that need improvement. A significant number of patients could not be located for study at the FLS. We consider this to be one area of improvement the unit should pursue. Coordination with the primary care doctor could improve the induction rate of patients, who otherwise reject such services due to lack of information, or who are diagnosed by other means. Our work reflects the difficulties of including the maximum number of patients in a secondary fracture prevention program. Despite the efforts we make to have an excellent FLS, we are not able to achieve 50% of patients in secondary prevention in the overall health area. This study is an internal audit that makes us reflect on ways to continue improving in the future. Our unit could have the capacity to include up to 600 new patients per year in the current format. To ensure that in the health area secondary prevention reaches, for example, 70% of fragility fractures, we should plan about other parallel actions such as training of medical doctors and a warning in the electronic medical record. In addition, for hip fracture, it is more effective in our experience to identify and recommend treatment during admission than through other recruitment methods [30]. Apart from initiatives undertaken by an FLS, every doctor who cares for a patient with a fragility fracture should consider secondary prevention strategies, including the use of drugs that have been proven effective.

This study did not include patients who had not been attended by our hospital’s emergency department, nor did it include patients with vertebral fracture, as has already been mentioned, or those with private insurance or patient cooperatives who were treated at private or subsidized centers. This last circumstance is very infrequent in our health area and did not significantly affect the results.

The greatest strength of this study lies in the large number of patients analyzed. It thus reflects what is happening in the northern area of Gran Canaria.

In conclusion, despite not all patients with fragility fractures are captured in a secondary prevention program, active treatment is prescribed more often in FLS units (increases from 20.4% in standard care to 40.7% with the implementation of the FLS in our health area) 
